# Dependence of Tensile Ductility and Impact Toughness on Constituent Particles in 2014 Aluminum Alloy

**DOI:** 10.3390/ma19122665

**Published:** 2026-06-21

**Authors:** Geng Chen, Fang Li, Sijun Chen, Songyi Chen, Kanghua Chen

**Affiliations:** 1 Light Alloy Research Institute, Central South University, Changsha 410083, China; chenundgeng@163.com (G.C.); sijunchen@163.com (S.C.); 2 Powder Metallurgy Research Institute, Central South University, Changsha 410083, China; lifanghonor@hnu.edn.cn

**Keywords:** 2014 aluminum alloy, impact toughness, constituent particles, mathematical model

## Abstract

In contemporary engineering applications, deficiencies in dynamic mechanical properties, particularly impact toughness, are the leading cause of fracture incidents. Consequently, inadequate dynamic mechanical properties have emerged as the primary constraint limiting the further commercial application of precipitation-strengthened high-strength aluminum (Al) alloys, exemplified by the 2014 aluminum alloy. Since the dynamic mechanical properties of the 2014 wrought aluminum alloy are fundamentally governed by the decohesion and cracking of coarse second-phase constituent particles, it is necessary to quantify the correlation between microstructure and mechanical properties. Meanwhile, the size and volume fraction of constituent particles are largely dictated by the concentration of main and impurity alloying elements. Experimental results revealed that the volume fraction of coarse constituents increased with increasing Cu, Si, and Fe content, and that tensile ductility and impact toughness decreased following an inverse exponential relationship with the volume fraction of constituents. The aim of this study is to establish a quantitative relation to correlate the characteristics of coarse constituents with the tensile ductility and impact toughness of the 2014 aluminum alloy. A mathematical model was developed by regarding the coarse constituents as ellipsoidal inclusions. Their volume fraction and aspect ratio were considered in the model. Model predictions show broad agreement with experimental data. These properties are more sensitive to the volume fraction when it is low. Conversely, a larger aspect ratio leads to higher ductility and toughness. The sensitivity is also greater at a small aspect ratio. The model further indicates that reducing the volume fraction when it is high yields limited improvement, whereas further reduction at a low volume fraction leads to significant enhancement of ductility and toughness. This study correlates coarse constituent characteristics with tensile ductility and impact toughness quantitatively, and provides a theoretical framework for predicting and optimizing the mechanical properties of 2014 aluminum alloy.

## 1. Introduction

Precipitation-strengthened aluminum alloys, such as the 2014 aluminum alloy, find extensive use across various industrial fields including aerospace, automobile, and welding because of their remarkable specific strength, formability, weldability, and thermal stability [1,2,3,4,5,6,7,8,9]. Until the end of the 20th century, researchers and companies primarily prioritized the improvement of strength over that of toughness [10,11,12,13,14]. Nevertheless, inadequate static/quasi-static strength or rigidity gives rise to much fewer structural failures nowadays. Instead, a deficiency in dynamic properties, impact toughness, for instance, induces the highest percentage of fractures. Consequently, relatively lower dynamic mechanical properties have emerged as the most dominant constraint to the commercial application of precipitation-strengthened high-strength aluminum alloys. Therefore, a large number of studies [15,16,17,18,19,20,21,22], aimed at withstanding higher quasi-static and dynamic loads, were dedicated to examining the relationship between tensile ductility, fracture toughness, impact toughness and metallurgical factors. From previous works, these metallurgical factors could be concluded as: (1) the volume fraction, dimension, and aspect ratio of second-phase particles; (2) strain localization; (3) grain size and morphology; and (4) precipitation free zones (PFZs). Second-phase particles are the dominant factor influencing both quasi-static and dynamic mechanical properties of high-strength aluminum alloys, since strain localization, grain morphology, and PFZs are essentially associated with them. This indicates the non-independence of the latter three factors. Therefore, only second-phase particles are taken into account in this study.

Second-phase particles in the precipitation-strengthened 2014 aluminum alloy can be categorized into three main types: micrometer-scale ellipsoidal constituents visible under an optical microscope, submicron spherical dispersoids, and nanometer-scale disk or needle-shaped precipitations, the latter two of which require observation through a transmission electron microscope. The presence of coarse constituents is primarily attributed to Fe and Si impurities or an excess of main alloying elements like Cu. Depending on the fabrication process, the volume fraction and size of these coarse constituents range normally from 1% to 5% and 5 to 30 μm [23,24,25]. Coarse constituents exert significant influence on the tensile ductility, strength, and toughness of the 2014 alloy since they play a crucial role in crack initiation. Dispersoids normally derive from the inclusion of minor elements such as Mn, Zr, etc., and form during the homogenization process, generally exhibit a volume fraction ranging from 0.05% to 0.2% and dimensions spanning from 20 to 500 nm [26,27,28,29]. The mechanical properties of the 2014 alloy are also shaped by dispersoids, which influence the growth and merging of initial voids, thus exerting an impact on the tensile ductility and impact toughness of the alloy. Nanometer-sized precipitations emerge within the 2014 alloy during the aging treatment. These exceedingly tiny second phases regulate dislocation mobility, thereby affecting the fracture process of the alloy indirectly [30,31,32,33].

However, due to their tendency to cleave or separate from the matrix, brittle constituents serve not only as common initiation sites for cracks, but also as pathways through which cracks could propagate more easily than through the matrix. Consequently, coarse constituents, which are theoretically detrimental to ductility and toughness, play the most important role in determining the properties of the 2014 aluminum alloy. Furthermore, experimental results have shown that reducing the percentage of Fe and Si significantly decreases the volume fraction of constituents within high-strength aluminum alloys, leading to simultaneous improvements in both tensile ductility and toughness [34,35,36,37]. In contrast, excessive Cu addition results in higher volume fraction of constituents, resulting in lower strength, tensile ductility, and toughness [38,39]. This principle has been employed to develop high-strength aluminum alloys such as 2014, 2214, 7075, and 7475. Besides experimental studies, many theoretical models have also been established to describe the relationship between tensile ductility, impact toughness, fracture toughness, and factors such as volume fraction, mean diameter, and interparticle spacing of constituents, either qualitatively or quantitatively [40,41,42,43,44,45]. Several empirical equations were proposed to illustrate the relationship between impact toughness and fracture toughness of high-strength steels [46,47,48]. However, there is currently no theory that comprehensively considers the relationship between constituents and mechanical properties of 2014 aluminum alloy, including tensile ductility, fracture toughness, and impact toughness at the same time. Furthermore, the inherent relationship between fracture toughness and impact toughness has not been deeply discussed. Although there is an existing experimental method to obtain valid $K_{IC}$ values, the fabrication of $K_{IC}$ samples is still complex, and the $K_{IC}$ tests also cost a lot of time and money. Establishing a correlation between Charpy V-notch (CVN) impact toughness and fracture toughness could be useful to simplify the prediction of fracture toughness.

In this study, based on the assumption that coarse constituents can be regarded as microcracks, and by analyzing the differences and similarities between fracture toughness and impact toughness, we proposed a quantitative equation relating fracture toughness and impact toughness while establishing an integrated model to elucidate the influence of coarse constituents on the tensile ductility and impact toughness of the 2014 high-strength aluminum alloy. Mechanical properties of the 2014 extruded bars with different Cu, Si, and Fe contents, namely bars containing different volume fractions and sizes of constituents, were determined and the dimensions, volume fraction, and aspect ratio of constituents and precipitations were also calculated to validate this model.

## 2. Materials and Methods

### 2.1. Material Preparation

In the present work, 2014 aluminum alloy extruded bar was adopted as the target material. Alloy ingots were prepared by general smelting process. Same heat treatments were performed on the alloys with different compositions to exclude the influence of heat treatments on tensile ductility and impact toughness. Their chemical compositions are shown in Table 1. The bulk compositions of the samples were determined by a SPEC-MAX optical emission spectrometer (Spectro Analytical Instruments Co., Ltd., Kleve, Germany), with K2014 certified reference material employed for calibration. Cast ingots were homogenized at 495 ± 2 °C for 24 h, and cooled in air. After homogenization, the cast ingots were machined to a diameter of 98 mm. Hot extrusion was performed at 440 °C. The cross-section was transformed from a circular to a rectangular (12 mm in thickness and 60 mm in width) geometry with a deformation ratio of approximately 10:1. After hot extrusion, extruded bars underwent a solid solution treatment at 503 ± 2 °C for 3 h, followed by an immediate water quench to room temperature. Subsequently, the alloys were subjected to artificial aging at 160 ± 2 °C for 16 h (T6).

Tensile tests were carried out at room temperature on an Instron 8801 axial servo-hydraulic fatigue testing machine (Instron Co., Ltd., Norwood, MA, USA). The total length, gauge length, width, and thickness of specimens are 80 mm, 30 mm, 6 mm, and 2 mm, respectively. A 25 mm extensometer was attached to specimens and the tensile speed was 2 mm/min.

The dimensions of the Charpy impact toughness test specimens are 55 mm in length, 10 mm in width and 10 mm in thickness. A 45° 2 mm deep V-notch at the center along the extrusion direction as pre-notch. The tensile and impact toughness results in this work represent at least the average of three parallel tests.

Grain morphology was observed by a Leica DM2700M/DFC450 optical microscope (Leica Microsystems GmbH, Wetzlar, Germany). The distribution of constituents, fracture morphology and energy-dispersive spectroscopy (EDS) analysis were examined by a ZEISS Merlin Compact field emission scanning electron microscope (Carl Zeiss AG, Oberkochen, Germany). Transmission electron microscope (TEM) images of precipitate characteristics were collected by a Titan G2 60-300 microscope operated at 300 kV (Thermo Fisher Scientific, Waltham, MA, USA). The average size of precipitation, average size and aspect ratio of constituents were counted by Image J (version 2.16.0). These data were averaged from at least five TEM or SEM images for each condition, and dimensions of at least 200 precipitates and 100 constituents were summarized. The mesoscale constituents can be regarded as pre-existing microcracks, whereas the nanoscale precipitates primarily influence the plastic deformation process through their pinning effect on dislocations, thereby affecting the elongation and impact toughness of the alloy. TEM samples were prepared by twin-jet electro-polishing at −25 °C with a current of 90~100 mA. The electrolyte was a mixture of 30% nitric acid and 70% methanol solution.

Since the constituents are irregular in shape, an effective particle diameter $d_{c}$ was determined as:

(1) $$d_{c}=\sqrt{d_{1}d_{2}}$$

where $d_{1}$ and $d_{2}$ are the smallest and largest dimensions of a constituent. The effective constituent radius $\bar{r_{c}}$ of n particles is given by:

(2) $$\bar{r_{c}}=\frac{\sum_{i=1}^{n}\sqrt{d_{1}d_{2}}}{2n}$$

Constituent volume fraction measurements were made at a magnification of 1000× using Image J. Six random views on each of three specimens were examined. The distribution of the constituents within each alloy was assumed to be random. Therefore, the volume fraction $f_{c}$ was taken as the ratio of the area of constituent particles to the total area of an image. Constituents smaller than 5 μm were not taken into calculation. Constituents smaller than 5 μm occupy fewer than 20 pixels in the SEM images at a magnification of 1000× and lead to large statistical errors in the size measurement. Meanwhile, excluding such small constituents has a negligible effect on the measured volume fraction of the constituents. Higher magnification results in too few coarse constituents per image for reliable statistics, while a lower magnification requires the exclusion of even larger constituents, which influences the statistical results substantially.

### 2.2. Tensile Ductility Model

**Figure 1:**
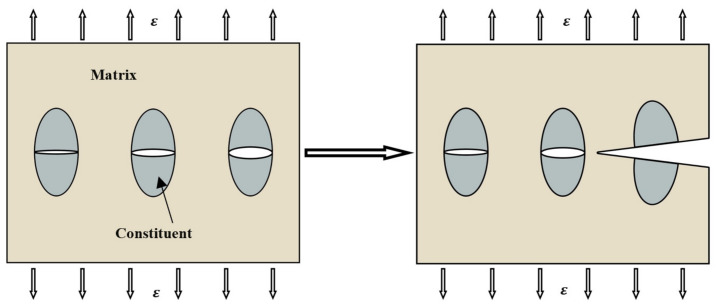
Schematic diagram of constituents acting as crack initiation sites and propagation path.

Previous studies proved that coarse brittle constituents fracture during deformation [23,24,25,49]. Compared to the α-Al matrix, coarse constituent particles typically exhibit much higher strength and significantly lower plasticity. The discrepancies in crystal structure and crystallographic orientation between the matrix and microscale constituent particles result in pronounced plastic strain incompatibility during deformation. Consequently, intense strain localization at the micron-scale coarse particles triggers transgranular particle cleavage and interface decohesion. These localized fractures not only serve as the primary sites for microcrack nucleation, but also facilitate rapid crack coalescence and premature fracture. Therefore, it is reasonable to regard constituents as microcracks (see Figure 1).

**Figure 2:**
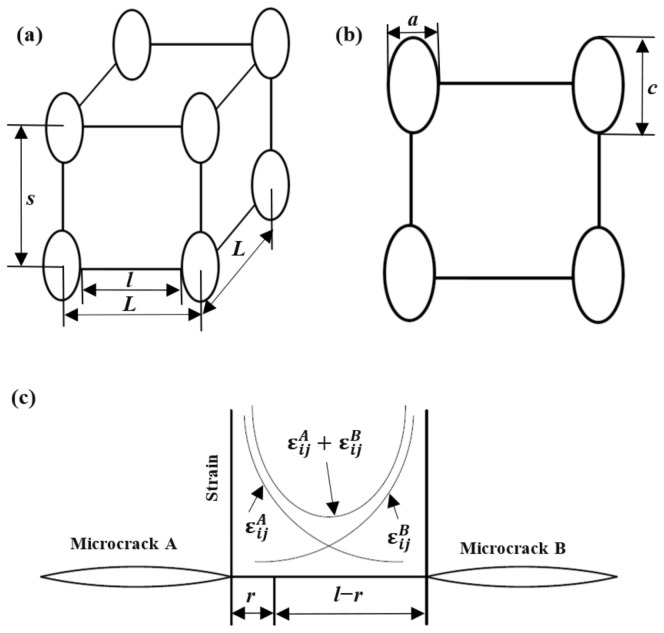
Isometric (**a**) and front (**b**) views of the schematic “unit cell” of constituents, and the strain distribution in the transition zone between two neighboring microcracks (**c**).

As shown in Figure 2a, the ellipsoidal constituents have two equal short axes, both of length *a*, and a long axis of length *c*. *L* is the center-to-center distance between two coarse constituents, while l is the transverse spacing between them, and s is the longitudinal spacing. The volume fraction of the coarse constituents is obtained as [50]:

(3) $$f_{c}=\frac{V}{L^{2}s}$$

where *V* is the volume of constituents. Since L=l+2a, *l* can be obtained as:

(4) $$l=2a\left(\zeta^{-1/2}-1\right)$$

where

(5) $$\zeta =\frac{f_{c}s}{\pi \lambda }$$

and

(6) $$\lambda =\frac{V}{4\pi a^{2}}=\frac{c}{3}$$

The plastic strain concentration in the transition zone between two coarse constituents/microcracks is shown in Figure 2c. The strain tensor at a distance r from microcrack A is given by [51]:

(7) $$\varepsilon_{ij}^{A}=\alpha \varepsilon_{y}{\left[\frac{J}{\alpha \varepsilon_{y}\sigma_{y}Ir}\right]}^{\frac{1}{1+n}}{\tilde{\varepsilon }}_{ij}\left(\theta \right)$$

where J is the *J*-integral, $\varepsilon_{y}$ is yield strain, $\sigma_{y}$ is yield stress, *n* is the strain hardening exponent, α is a constant in Ramberg–Osgood constitutive relation. *I* and ${\tilde{\varepsilon }}_{ij}\left(\theta \right)$ are normalized parameters in the HRR field [51,52]. The strain tensor due to microcrack B is:

(8) $$\varepsilon_{ij}^{B}=\alpha \varepsilon_{y}{\left[\frac{J}{\alpha \varepsilon_{y}\sigma_{y}I\left(1-r\right)}\right]}^{\frac{1}{1+n}}{\tilde{\varepsilon }}_{ij}\left(\theta \right)$$

The total plastic strain is obtained by adding Equations (7) and (8) together:

(9) $$\varepsilon_{ij}=\alpha \varepsilon_{y}{\left[\frac{QJ}{\alpha \varepsilon_{y}\sigma_{y}Ir}\right]}^{\frac{1}{1+n}}{\tilde{\varepsilon }}_{ij}\left(\theta \right)$$

where *I* is a function of *n* [40]:

(10) $$I=10.3\sqrt{0.13+n}-4.8n$$

and

(11) $$Q={\left[1+{\left(\frac{r}{l-r}\right)}^{\frac{1}{1+n}}\right]}^{1+n}$$

is the strain increment close to the microcrack tip.

Since the *J*-integral given by [32] is:

(12) $$J\approx J_{p}=\frac{h}{1-\nu^{2}}\left(\frac{\varepsilon_{p}}{\varepsilon_{e}}\right)J_{e}$$

where $\varepsilon_{e}$ and $\varepsilon_{p}$ are elastic and plastic strains. *h* is also a function of *n* [50]:

(13) $$h=\frac{3}{2\sqrt{1+3n}}$$

The elastic *J*-integral $J_{e}$ is [53,54]:

(14) $$J_{e}=\frac{0.405h\sigma_{app}\pi a}{E}\left(\frac{\varepsilon_{p}}{\varepsilon_{e}}\right)$$

where $\sigma_{app}$ is the applied stress, and *E* is Young’s modulus. Combining Equation (14) with Ramberg–Osgood equation:

(15) $$\frac{\varepsilon_{p}}{\varepsilon_{y}}=\alpha {\left(\frac{\sigma }{\sigma_{y}}\right)}^{\frac{1}{n}}$$

with $\sigma =\sigma_{app}$, $\varepsilon_{e}=\sigma /E$, and $\varepsilon_{y}=\sigma_{y}/E$. The *J*-integral is:

(16) $$J=\frac{0.405\pi h\sigma_{y}a{\left(\varepsilon_{p}\right)}^{n+1}}{{\left(\alpha \varepsilon_{y}\right)}^{n}}$$

Substituting Equation (16) into Equation (9), the macrocritical plastic strain is:

(17) $$\varepsilon_{p}=\frac{1}{{\tilde{\varepsilon }}_{n}\left(\theta \right)}{\left[\frac{I}{0.405\pi h}\right]}^{\frac{1}{n+1}}{\left[\frac{r}{a}\right]}^{\frac{1}{n+1}}\frac{{\tilde{\varepsilon }}_{r}}{\left[1+{\left(\frac{r}{l-r}\right)}^{\frac{1}{n+1}}\right]}$$

In Equation (17), ${\tilde{\varepsilon }}_{n}\left(\theta \right)$ is the effective value of the normalized coefficient ${\tilde{\varepsilon }}_{ij}\left(\theta \right)$ and is a constant for θ=0. ${\tilde{\varepsilon }}_{r}$ is the local effective strain at r, considering the relation between macrostrain $\varepsilon_{p}$ and the critical microstrain ($\tilde{\varepsilon }$) at the midpoint (r=l/2) of two neighboring microcracks. By defining $\varepsilon_{f}=\varepsilon_{p}$ $\lambda_{c}=L$, $r_{c}=a$, and defining $\tilde{\varepsilon }={\tilde{\varepsilon }}_{r}\left(r=l/2\right)$, the macrostrain to fracture $\varepsilon_{f}$ is obtained as:

(18) $$\varepsilon_{f}=\frac{1}{{\tilde{\varepsilon }}_{n}\left(\theta \right)}{\left[\frac{I}{0.405\pi h}\right]}^{\frac{1}{n+1}}{\left[\frac{\lambda_{c}}{2r_{c}}-1\right]}^{\frac{1}{n+1}}\frac{\tilde{\varepsilon }}{2}$$

$\tilde{\varepsilon }$ is influenced by the average length of the precipitates [40]. It is also the minimum strain required for the growth and coalescence of voids/microcracks.

### 2.3. Impact Toughness Model

According to the relationship between fracture toughness and critical fracture strain [55]:

(19) $$K_{IC}=\sqrt{\frac{2CE\varepsilon_{c}^{*}\sigma_{y}n^{2}}{\left(1-\nu^{2}\right)}}$$

where *C* is a constant ≈ 1/40, *E* is Young’s modulus, $\varepsilon_{c}^{*}$ is the critical local strain ahead of a main crack, $\sigma_{y}$ is the tensile yield strength, *n* is the strain hardening exponent and ν is Poisson’s ratio. It is widely known that $\varepsilon_{c}^{*}$ is a function of the volume fraction of constituents [56]:

(20) $$\varepsilon_{c}^{*}\;=f\left(\frac{1}{f_{c}}\right)$$

Earlier we derived the macrostrain to fracture $\varepsilon_{f}$ as Equation (18). From previous work [56], it has been suggested that, based on experimental results of commercial 2014 aluminum alloy [55], $\varepsilon_{c}^{*}$ in Equation (19) is probably half of the true strain ε under tensile conditions. Therefore, we can substitute $\varepsilon_{c}^{*}=\frac{1}{2}\varepsilon_{f}$ and Equation (16) into Equation (19) to obtain the final expression for fracture toughness:

(21) $$K_{IC}=A\sqrt{Bn^{2}\sigma_{y}{\left[\frac{\lambda_{c}}{2r_{c}}-1\right]}^{\frac{1}{n+1}}}$$

with

(22) $$A=\sqrt{\frac{CE}{2{\tilde{\varepsilon }}_{n}\left(\theta \right)}}$$

(23) $$B={\left[\left(1+3n\right)\left(5.39\sqrt{0.13+n}-2.53n\right)\right]}^{\frac{1}{n+1}}$$

For a cubic array, the volume fraction of constituents $f_{c}$ is a function of the mean radius and interparticle spacing of constituents, $r_{c}$ and $\lambda_{c}$, respectively [40]:

(24) $$f_{c}=2\pi p{\left(\frac{r_{c}}{\lambda_{c}}\right)}^{3}$$

where p is the aspect ratio of constituents. As a result, $\lambda_{c}/r_{c}$ is:

(25) $$\frac{\lambda_{c}}{r_{c}}={\left(\frac{2\pi p}{f_{c}}\right)}^{\frac{1}{3}}$$

$\lambda_{c}/r_{c}$, which is defined as the ratio of the inter-constituent spacing to the size of the coarse constituents, is the most critical parameter governing the ductility and toughness of the alloy. Physically, this parameter represents the degree of continuity of the coarse constituents. According to Equations (18) and (25), since $\lambda_{c}/r_{c}$ is proportional to the aspect ratio of the coarse constituents and inversely proportional to their volume fraction, the elongation and impact toughness are proportional to the aspect ratio of the coarse constituents and inversely proportional to their volume fraction. Other studies also proved this relation [40,49]. A larger aspect ratio results in a wider ligament between two coarse constituents, which improves the elongation and impact toughness of the alloy.

The general fracture process consists of plastic deformation, crack/void nucleation and growth, and final fracture. The most widely accepted fracture criterion is the generalized energy failure criterion; namely failure occurs as the energy absorbed reaches a critical value. It is well known that the crack propagates along the path of least resistance in materials, that is to say, along constituents. Thus, there is a criterion during crack initiation and propagation called the minimum energy density criterion. Both impact toughness [57] and fracture toughness [58] of materials obey this criterion. Meanwhile, although CVN and $K_{IC}$ are dynamic and quasi-static toughness, respectively, the effects of strain rate have been proved to be negligible for high-strength materials [59]. Based on the discussion above, there must be a quantitative equation between impact toughness and fracture toughness.

The Charpy V-Notch impact toughness consists of energy for crack initiation and propagation since the notch radius $\rho_{c}=0.25\;mm$. Fracture toughness consists only of the energy for crack propagation since the notch radius $\rho_{k}=0\;mm$. Meanwhile, for the age-hardened 2014 aluminum alloy, the elastic deformation energy is negligible compared to plastic deformation energy.

The Integration Is performed from the notch root (x=0) to the front of the plastic zone ($x=R_{p}$), and the boundary condition is that the local stress at ($x=R_{p}$) equals the yield strength $\sigma_{y}$ of the alloy. Accordingly, the energy integral can be expressed as:

(26) $$W=\int_{0}^{R_{p}}U\left(\sigma ,\varepsilon \right)Bdx$$

where *U* is the strain energy density, *B* is the thickness of specimen, and the upper integration limit $R_{p}$ is determined by the condition $\sigma \left(x=Rp\right)=\sigma_{y}$. $R_{p}$ depends on the local stress field and material properties and is commonly estimated using the classical Irwin plastic zone correction [60]:

(27) $$R_{p}=\frac{1}{\pi }{\left(\frac{K_{I}}{\sigma_{y}}\right)}^{2}$$

where $K_{I}$ is the stress intensity factor at the crack tip and $\sigma_{y}$ is the yield strength of the alloy. $R_{p}$ is negatively correlated with $\sigma_{y}$ and positively correlated with $K_{I}$. Since $\sigma_{y}$ is negatively correlated with $f_{c}$, $R_{p}$ is positively correlated with $f_{c}$.

Based on this consideration and the relationship between critical energy release rate $G_{IC}$ and critical stress field intensity $K_{IC}$:

(28) $$K_{IC}^{2}=\frac{EG_{IC}}{\left(1-\nu^{2}\right)}$$

as well as the linear relationship between $G_{IC}$ and *CVN* [61]:

(29) $$G_{IC}=\frac{CVN}{2}$$

there is a quantitative relationship between $K_{IC}$ and *CVN*:

(30) $$K_{IC}^{2}=k_{1}\left(CVN\right)$$

where $k_{1}$ is a constant. All impact toughness specimens in this study were prepared from the 2014 wrought aluminum alloy. The room temperature Charpy V-notch impact toughness tests were conducted using the same equipment with identical notch geometry, under the same constraint conditions, and at the same strain rate. Rasid [62] summarizes numerous quantitative fracture toughness–impact toughness relationships proposed by other researchers. Two of them in the intermediate temperature regime exhibit a direct proportionality between $K_{IC}^{2}$ and CVN. The predicted fracture toughness values showed only minor deviations from the experimental results when applied to the 2024 aluminum alloy. Therefore, it is reasonable to adopt the same constant $k_{1}$ for all alloy compositions investigated. Substituting Equation (6) into Equation (13), we obtain:

(31) $$CVN=\frac{A^{2}Bn^{2}\sigma_{y}{\left[\frac{\lambda_{c}}{2r_{c}}-1\right]}^{\frac{1}{n+1}}}{k_{1}}$$

We used one set of tensile ductility and impact toughness data as the reference, designated as ${\left(\varepsilon_{f}\right)}_{R}$ and ${\left(K_{IC}\right)}_{R}$, respectively. Normalization coefficients $R_{TD}$ (for ductility) and $R_{IT}$ (for impact toughness) were first defined as:

(32) $$R_{TD}=\frac{\varepsilon_{f}}{{\left(\varepsilon_{f}\right)}_{R}}$$

(33) $$R_{IT}=\frac{K_{IC}}{{\left(K_{IC}\right)}_{R}}$$

After obtaining the normalized results, the predicted curves of tensile ductility and impact toughness as functions of the relevant parameters were plotted by multiplying the normalized values by the respective normalization coefficients. Finally, all the remaining experimental data were used to validate the predictive ability of the model.

## 3. Results and Discussion

### 3.1. Multi-Phase Structure and Fracture Behavior of 2014 Alloy

**Figure 3:**
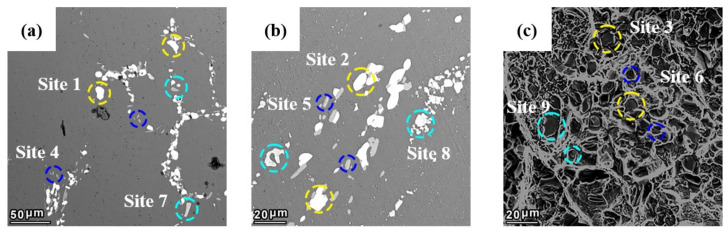
SEM images (several constituents marked with dash circle) of the (**a**) homogenized and (**b**) peak-aged microstructures, along with (**c**) fracture morphology of the peak-aged 2014 aluminum alloy strip.

Figure 3a–c represent the typical homogenized, T6 and fracture morphology of the 2014 aluminum alloy. After homogenization treatment, while dendritic segregation was largely dissolved, a significant number of coarse residual phases persisted along the grain boundaries (Figure 3a). During subsequent hot extrusion, these coarse phases were fragmented. After solid solution treatment, further dissolution of these phases occurred, resulting in a substantial reduction in their size compared to the homogenized state (Figure 3b). On the fracture morphology, cracked coarse constituent particles were located at the bottom of large dimples (Figure 3c). The surfaces of these dimples appeared smooth and exhibited characteristic cleavage features, indicating brittle fracture. This observation confirms that the coarse constituent particles acted as crack initiation sites for fracture.

The chemical compositions obtained from SEM-EDS point analysis at the circled locations in Figure 3 are summarized in Table 2. Most of the constituent particles in the 2014 aluminum alloy are Al_2_Cu, AlCuMgSi, and AlCuMgSiMnFe. Specifically, sites (1)~(3) correspond to the Al_2_Cu phase (marked by red dashed circles), sites (4)~(6) to the AlCuMgSi phase (green dashed circles), and sites (7)~(9) to the AlCuMgSiMnFe phase (light blue dashed circles). In Figure 3a,b, the brightest coarse particles are identified as Al_2_Cu. Most of them exhibit an elliptical morphology, with a minority being irregular in shape. However, all of them have rounded edges and no sharp corners. The dimensions of the Al_2_Cu phases are also larger than those of the other two phases. The relatively darker, smaller, and highly irregularly shaped constituent particles that tend to cluster together are identified as AlCuMgSiMnFe. The darkest and smallest constituents correspond to AlCuMgSi. As shown in Figure 3c, cracked constituent particles of all three phases are observed at the bottom of dimples on the fracture morphology, proving that all three kinds of constituents are brittle and can act as crack initiation sites in the matrix.

**Figure 4:**
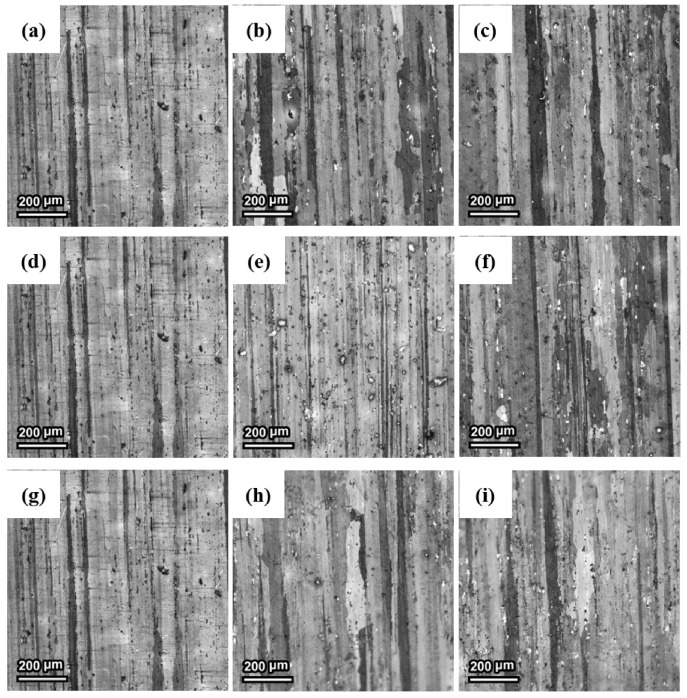
Grain morphology of (**a**) 4.00 Cu, (**b**) 4.27 Cu, (**c**) 4.59 Cu, (**d**) 0.74 Si, (**e**) 0.92 Si, (**f**) 1.04 Si, (**g**) 0.06 Fe, (**h**) 0.13 Fe, (**i**) 0.23 Fe peak-aged 2014 aluminum alloy.

Figure 4 shows the grain morphology images of all samples. The statistical results of the average grain size and average PFZ width are summarized in Table 3. The grains in all samples exhibit an elongated fibrous morphology without any characteristic of recrystallization. The statistical data in the table confirm that there are no significant differences in the average grain size or PFZ width among the samples.

**Figure 5:**
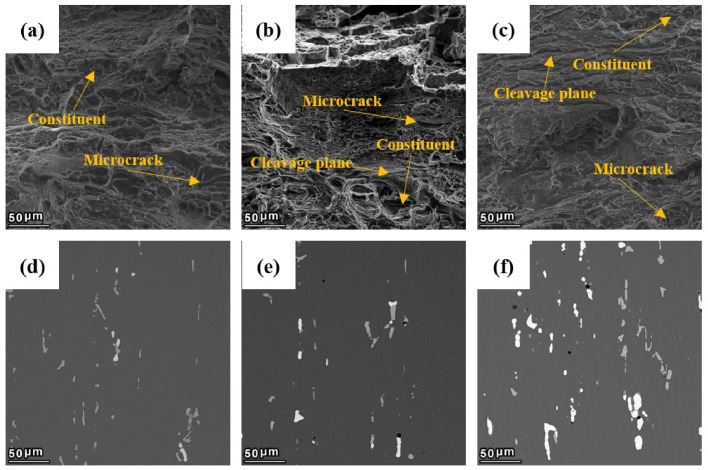
Fracture morphology of (**a**) 4.00 Cu, (**b**) 4.27 Cu, (**c**) 4.59 Cu and distribution of coarse constituent particles in peak-aged 2014 aluminum alloy with (**d**) 4.00 Cu, (**e**) 4.27 Cu, (**f**) 4.59 Cu content.

**Figure 6:**
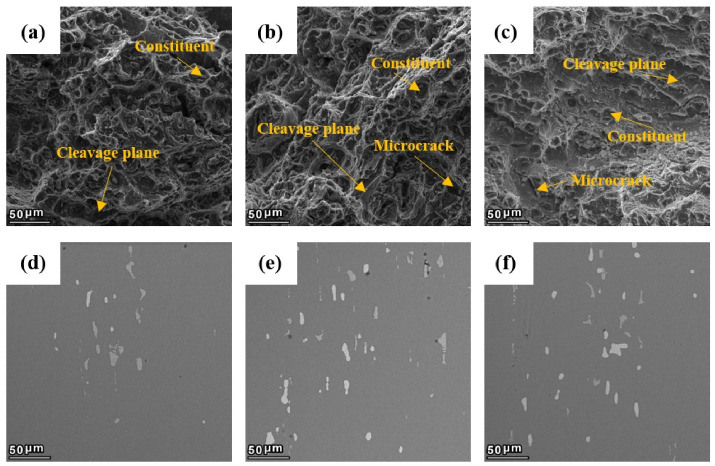
Fracture morphology of (**a**) 0.74 Si, (**b**) 0.92 Si, (**c**) 1.04 Si and distribution of coarse constituent particles in peak-aged 2014 aluminum alloy with (**d**) 0.74 Si, (**e**) 0.92 Si, (**f**) 1.04 Si content.

**Figure 7:**
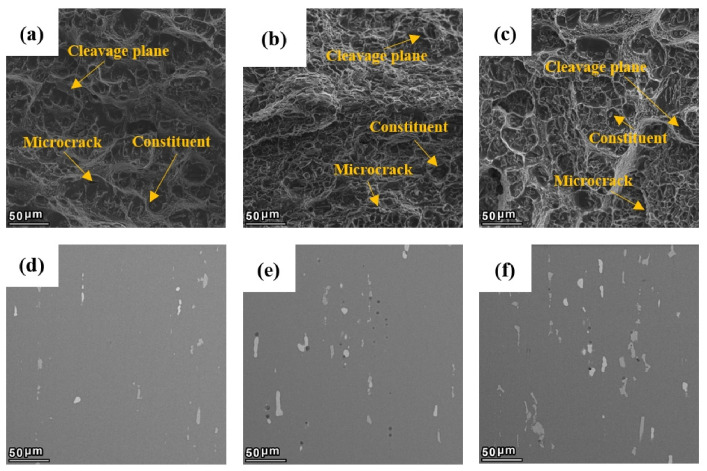
Fracture morphology of (**a**) 0.06 Fe, (**b**) 0.13 Fe, (**c**) 0.23 Fe and distribution of coarse constituent particles in peak-aged 2014 aluminum alloy with (**d**) 0.06 Fe, (**e**) 0.13 Fe, (**f**) 0.23 Fe. Content.

Figure 5, Figure 6 and Figure 7 illustrate the impact fracture morphologies and the distribution of coarse constituent particles in peak-aged specimens with varying contents of Cu, Si, and Fe. Qualitatively, as the contents of the three alloying elements increase, both the volume fraction and the average size of the three types of coarse constituent particles rise accordingly. The fracture surfaces of specimens with higher alloying element content appear relatively flatter compared to those with lower content. They also exhibit shallower and smaller dimples. Additionally, although all materials remain ductile and the fracture surface morphology is predominantly characterized by dimple fracture, a greater proportion of brittle fracture is observed on the fracture surfaces of specimens with higher alloying element content. In all fracture morphology images, cracked constituent particles are observed at the bottom of dimples. This suggests that the constituent particles act as crack initiation sites in all samples studied in this research.

**Figure 8:**
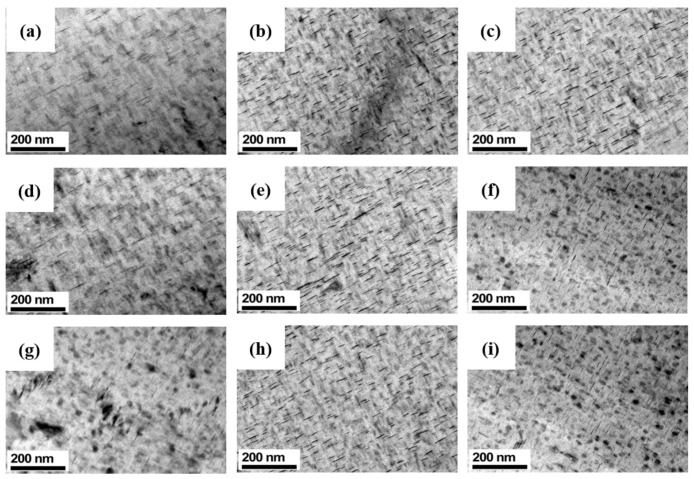
Bright field TEM images of 2014 aluminum alloys with varying Cu, Si, and Fe content: (**a**) 4.00 Cu, (**b**) 4.27 Cu, (**c**) 4.59 Cu, (**d**) 0.74 Si, (**e**) 0.92 Si, (**f**) 1.04 Si, (**g**) 0.06 Fe, (**h**) 0.13 Fe and (**i**) 0.23 Fe.

**Figure 9:**
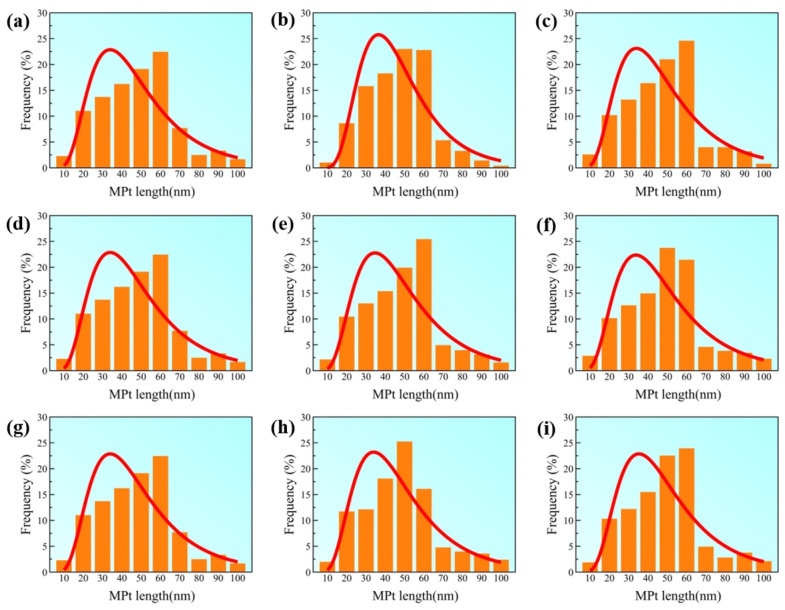
Statistical results of the precipitate size in 2014 aluminum alloys with varying Cu, Si, and Fe content: (**a**) 4.00 Cu, (**b**) 4.27 Cu, (**c**) 4.59 Cu, (**d**) 0.74 Si, (**e**) 0.92 Si, (**f**) 1.04 Si, (**g**) 0.06 Fe, (**h**) 0.13 Fe and (**i**) 0.23 Fe.

Figure 8 illustrates the bright-field (BF) TEM images showing the distribution and morphology of nano-scale precipitates within the grain interiors of peak-aged alloys with different Cu, Si, and Fe contents. It can be observed that in all 2014 wrought aluminum alloys, a large number of matrix precipitates (MPts) exhibit a needle-like shape and are distributed uniformly throughout the matrix. Figure 9 demonstrates the statistical results of precipitation size in the grain interiors. The size distributions of all samples follow a log-normal distribution. The corresponding statistical data for the volume fraction and average size of the $\theta^{\prime }$ phases are summarized in Table 3. These observations indicate that both the volume fraction and average dimensions of the $\theta^{\prime }$ phases remain relatively invariant despite the variation in element content. Therefore, in this study, the contributions of the grain size, precipitation size, and PFZ to variations in tensile ductility and impact toughness can be considered negligible. The parameter $\tilde{\varepsilon }$ in Equation (18) can be approximated as a constant and allows the predictive model to focus exclusively on the influence of the coarse constituent particles.

**Figure 10:**
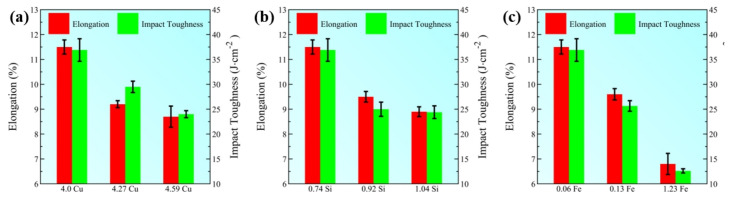
Ultimate elongation and impact toughness of 2014 aluminum alloys with varying (**a**) Cu, (**b**) Si, and (**c**) Fe content.

Elongation and *CVN* ($a_{k}$) of all samples are shown in Figure 10. Both the ultimate elongation and impact toughness decrease as the contents of Cu, Si, and Fe increase. The volume fraction $f_{c}$, average size, and aspect ratio *p* of constituent particles, as well as the elongation and impact toughness, are also listed in Table 4. The volume fraction of the constituent particles shows a good correlation with the increase in alloying element content. However, the aspect ratio of constituents remains approximately the same. The alloy composition primarily affects the average size and volume fraction of the coarse constituents, while its influence on their aspect ratio is small. The size of the coarse constituents influences the mechanical properties indirectly through the composite parameter $\lambda_{c}/r_{c}$. Physically, this parameter represents the degree of continuity of the coarse constituents. $r_{c}$ is therefore not an independent parameter, or rather, the influence of $r_{c}$ on tensile ductility and impact toughness is reflected by the volume fraction and aspect ratio of constituents. In our model, the effects of the volume fraction and aspect ratio of the coarse constituents are considered. These two parameters are independent and directly correlated with the ductility and toughness of the alloy.

Cu promotes the formation of the coarse constituent Al_2_Cu, Si promotes the coarse constituent AlCuMgSi/AlCuMgSiMnFe, and Fe promotes the coarse constituent AlCuMgSiMnFe. All three types of coarse constituents are hard and brittle. Experimental results indicate that these coarse constituents fracture at a certain point during the plastic deformation, rather than remaining intact due to interfacial debonding or other factors. Under the continuity assumption, the strain within the coarse constituents is approximately equal to that in the surrounding matrix. Owing to their extremely poor ductility, they fracture at a very early stage of deformation. Both experimental results and theoretical analysis suggest that the constituents in the 2014 wrought aluminum alloy can be regarded as pre-existing microcracks. Consequently, the nucleation and growth of microvoids at the coarse constituents is the primary mechanism by which these particles influence the fracture process of the alloy.

### 3.2. Influence of Constituents on Tensile Ductility and Impact Toughness

The values, expressions, physical meanings, and methods of determination for all parameters used in the model are listed in Table 5.

**Figure 11:**
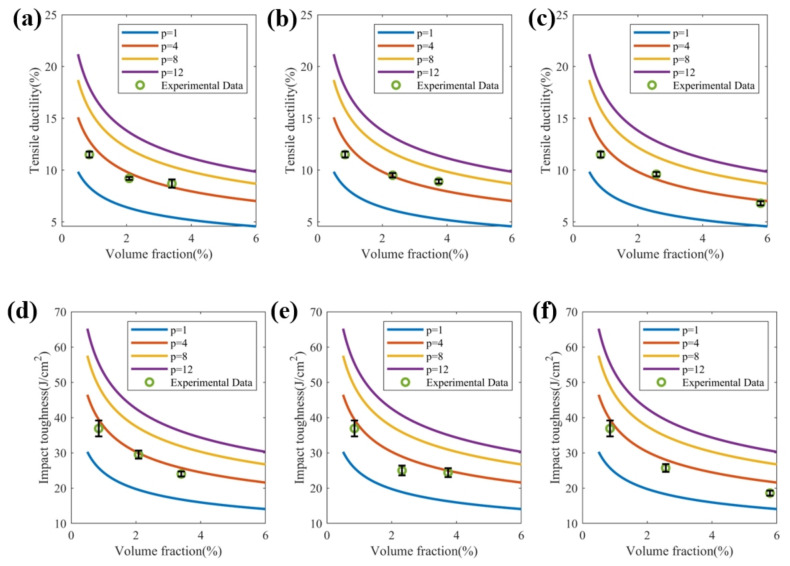
Comparison between experiment and theory: Tensile ductility of samples with different (**a**) Cu content, (**b**) Si content, and (**c**) Fe content. Impact toughness of samples with different (**d**) Cu content, (**e**) Si content, and (**f**) Fe content.

As shown in Figure 11, experimental data of the tensile ductility and impact toughness of 2014 aluminum alloys containing different Cu, Si, and Fe contents fit well with the theoretical predictions (red line). It can be concluded that the volume fraction and aspect ratio of constituents have a great influence on the tensile ductility and impact toughness of 2014 aluminum alloy. According to the experimental results, the volume fraction of coarse constituents increases with increasing Cu, Si, and Fe contents. A higher volume fraction of constituents consequently leads to a decrease in tensile ductility and impact toughness.

**Figure 12:**
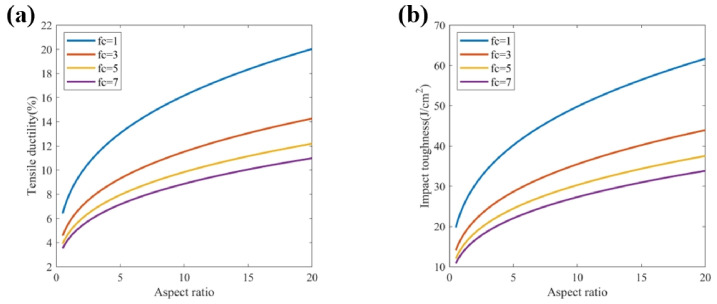
Model prediction of the effect of aspect ratio on tensile ductility and impact toughness (**a**) tensile ductility, (**b**) impact toughness.

The theoretical model predicts that the elongation and impact toughness of the alloy decrease continuously with increasing volume fraction of coarse constituents. In addition, these properties are more sensitive to the volume fraction when the volume fraction is low. The theoretical model also predicts (Figure 12) that the elongation and impact toughness of the alloy increase continuously with increasing aspect ratio of the coarse constituents, with a higher sensitivity at a small aspect ratio. This indicates that when the volume fraction of coarse constituents is high, reducing it does not improve tensile ductility and toughness effectively, while further reduction at low volume fraction can lead to a significant improvement.

Moreover, according to Equation (25), for a given volume fraction of constituents, a larger aspect ratio means a greater $\lambda_{c}/r_{c}$ in Equations (18) and (19), and thus higher tensile ductility and impact toughness. Conversely, for a given aspect ratio, a higher volume fraction reduces $\lambda_{c}/r_{c}$ and leads to lower tensile ductility and impact toughness.

## 4. Conclusions

The dependence of tensile ductility and impact toughness on constituents in the 2014 aluminum alloy was studied in this work. Briefly, the conclusions are as follows:

With increasing Cu, Si, and Fe contents, the volume fraction and average size of constituents increase rapidly while the variation in aspect ratio is negligible. Meanwhile, the volume fraction, average size, dimension distribution of precipitations, as well as the average grain size, and PFZ width, remain almost the same since the already-saturated matrix is unable to gain a higher degree of supersaturation.

With increasing volume fraction of constituents, the ultimate elongation and impact toughness of the 2014 aluminum alloy decrease. Experimental data of tensile ductility and impact toughness of the samples containing different Cu, Si, and Fe contents fit well with theoretical predictions. When the volume fraction is high, the coarse constituents may become interconnected, which violates the assumption of isolated ellipsoidal inclusions. Under such conditions, the model predictions may therefore deviate from experimental observations.

The key parameter that influences the tensile ductility and impact toughness of the 2014 aluminum alloy is the ratio of the inter-particle spacing to the radius of constituents $\lambda_{c}/r_{c}$. This parameter has a positive correlation with the aspect ratio of constituents and a negative correlation with the volume fraction of constituents.

This study correlates coarse constituent characteristics with tensile ductility and impact toughness quantitatively, and provides a theoretical framework for predicting and optimizing the mechanical properties of 2014 aluminum alloy and other age-hardened aluminum alloys.

## Figures and Tables

**Table 1:** Chemical compositions (wt.%) of 2014 extruded strips.

Samples	Cu	Mg	Si	Mn	Ti	Fe	Al
4.00 Cu	4.00	0.38	0.74	0.69	0.03	0.06	Bal.
4.27 Cu	4.27	0.39	0.83	0.75	0.02	0.06	Bal.
4.59 Cu	4.59	0.40	0.79	0.70	0.02	0.06	Bal.
0.74 Si	4.00	0.38	0.74	0.69	0.03	0.06	Bal.
0.92 Si	4.18	0.44	0.92	0.69	0.02	0.05	Bal.
1.04 Si	4.23	0.39	1.04	0.69	0.02	0.04	Bal.
0.06 Fe	4.00	0.38	0.74	0.69	0.03	0.06	Bal.
0.13 Fe	4.13	0.30	0.76	0.64	0.02	0.13	Bal.
0.23 Fe	4.06	0.30	0.81	0.71	0.02	0.23	Bal.

**Table 2:** Chemical compositions (at. %) of constituents in homogenized ingot and strip after supersaturation or artificial aging.

Site Number	Al	Cu	Mg	Si	Mn	Fe
1	58.26	41.74	-	-	-	-
2	71.93	28.07	-	-	-	-
3	58.54	41.11	-	0.35	-	-
4	33.66	11.56	33.55	21.24	-	-
5	25.79	19.68	34.97	19.56	-	-
6	45.64	9.73	27.46	17.16	-	-
7	72.10	5.20	0.33	11.25	7.69	3.43
8	74.83	4.23	0.43	9.83	7.30	3.37
9	71.48	5.06	0.88	10.05	8.72	3.80

**Table 3:** Average grain size, average PFZ width, as well as area fraction and average size of precipitations in 2014 aluminum alloys with different Cu, Si, and Fe contents.

Samples	Average Grain Size (μm)	Average PFZ Width (nm)	Area Fraction of MPt (%)	Average MPt Size (nm)
4.0 Cu	59.64 ± 9.23	105.84 ± 6.23	6.62 ± 1.17	47.83 ± 8.64
4.27 Cu	58.83 ± 10.09	109.27 ± 7.69	6.89 ± 1.02	48.52 ± 8.13
4.59 Cu	60.28 ± 9.72	102.65 ± 4.35	7.13 ± 1.14	48.61 ± 6.92
0.74 Si	59.64 ± 9.23	105.84 ± 6.23	6.62 ± 1.17	47.83 ± 8.64
0.92 Si	60.49 ± 10.52	101.42 ± 7.21	6.99 ± 1.31	44.07 ± 8.29
1.04 Si	59.31 ± 10.47	111.35 ± 5.98	6.47 ± 1.15	47.12 ± 8.47
0.06 Fe	59.64 ± 9.23	105.84 ± 6.23	6.62 ± 1.17	47.83 ± 8.64
0.13 Fe	60.11 ± 9.96	107.58 ± 6.43	6.30 ± 1.04	48.10 ± 7.58
0.23 Fe	58.26 ± 9.19	107.16 ± 6.86	6.88 ± 1.08	48.94 ± 6.21

**Table 4:** Tensile ductility, impact toughness and characteristics of constituents of 2014 aluminum alloys with different Cu, Si, and Fe contents.

Samples	δ (%)	$a_{K}$ (J × cm^−2^)	$f_{c}$ (%)	Average Size (μm)	k
4.0 Cu	11.5 ± 0.28	36.9 ± 2.26	0.85 ± 0.07	14.76 ± 1.15	3.89 ± 0.14
4.27 Cu	9.2 ± 0.14	29.5 ± 1.13	2.08 ± 0.19	19.99 ± 1.84	3.82 ± 0.21
4.59 Cu	8.7 ± 0.39	24.0 ± 0.71	3.40 ± 0.24	25.71 ± 2.91	3.94 ± 0.17
0.74 Si	11.5 ± 0.28	36.9 ± 2.26	0.85 ± 0.07	14.76 ± 1.15	3.89 ± 0.14
0.92 Si	9.5 ± 0.21	25.0 ± 1.41	2.32 ± 0.23	20.87 ± 2.09	4.03 ± 0.15
1.04 Si	8.9 ± 0.20	24.4 ± 1.27	3.74 ± 0.16	25.01 ± 2.43	3.93 ± 0.19
0.06 Fe	11.5 ± 0.28	36.9 ± 2.26	0.85 ± 0.07	14.76 ± 1.15	3.89 ± 0.14
0.13 Fe	9.6 ± 0.23	25.7 ± 1.06	2.57 ± 0.32	19.23 ± 2.26	4.07 ± 0.23
0.23 Fe	6.8 ± 0.42	12.6 ± 0.45	5.79 ± 0.48	26.09 ± 2.84	4.01 ± 0.18

**Table 5:** Parameters used in the mathematical models.

Parameter	Value	Description
$\varepsilon_{f}$	Calculated	Tensile ductility
${\tilde{\varepsilon }}_{n}\left(\theta \right)$	Constant	Fitted parameter
*I*	$10.3\sqrt{0.13+n}-4.8n$	Function of *n*
*h*	$3/\left(2\sqrt{1+3n}\right)$	Function of *n*
*n*	0.08	Strain hardening exponent
$\lambda_{c}$	Measured	Inter spacing of constituents
$r_{c}$	Measured	Dimension of constituents along crack propagation direction
$\lambda_{c}/r_{c}$	Calculated	Degree of continuity of the constituents
$\tilde{\varepsilon }$	Constant	Critical microstrain at the midpoint of two neighboring microcracks
*p*	Measured	Aspect ratio of constituents
$f_{c}$	Measured	Volume fraction of constituents
*CVN*	Calculated	Impact toughness
A	$\sqrt{CE/\left(2{\tilde{\varepsilon }}_{n}\left(\theta \right)\right)}$	Composite parameter
B	${\left[\left(1+3n\right)\left(5.39\sqrt{0.13+n}-2.53n\right)\right]}^{\frac{1}{n+1}}$	Composite parameter
$\sigma_{y}$	Measured	Yield strength
$k_{1}$	Constant	Fitted parameter

## Data Availability

The original contributions presented in this study are included in the article. Further inquiries can be directed to the corresponding authors.
